# Serum and glucocorticoid-regulated kinase 1: Structure, biological functions, and its inhibitors

**DOI:** 10.3389/fphar.2022.1036844

**Published:** 2022-11-15

**Authors:** Hyunsoo Jang, Youngjun Park, Jaebong Jang

**Affiliations:** ^1^ College of Pharmacy, Korea University, Sejong, South Korea; ^2^ Laboratory of Immune and Inflammatory Disease, College of Pharmacy, Jeju Research Institute of Pharmaceutical Sciences, Jeju National University, Jeju, South Korea; ^3^ Interdisciplinary Graduate Program in Advanced Convergence Technology and Science, Jeju National University, Jeju, South Korea

**Keywords:** SGK1, kinase inhibitor, ion channel, cancer, T cell modulation

## Abstract

Serum and glucocorticoid-regulated kinase 1 (SGK1) is a serine/threonine kinase belonging to the protein kinase A, G, and C (AGC) family. Upon initiation of the phosphoinositide 3-kinase (PI3K) signaling pathway, mammalian target of rapamycin complex 2 (mTORC2) and phosphoinositide-dependent protein kinase 1 (PDK1) phosphorylate the hydrophobic motif and kinase domain of SGK1, respectively, inducing SGK1 activation. SGK1 modulates essential cellular processes such as proliferation, survival, and apoptosis. Hence, dysregulated SGK1 expression can result in multiple diseases, including hypertension, cancer, autoimmunity, and neurodegenerative disorders. This review provides a current understanding of SGK1, particularly in sodium transport, cancer progression, and autoimmunity. In addition, we summarize the developmental status of SGK1 inhibitors, their structures, and respective potencies evaluated in pre-clinical experimental settings. Collectively, this review highlights the significance of SGK1 and proposes SGK1 inhibitors as potential drugs for treatment of clinically relevant diseases.

## 1 Introduction

Serum and glucocorticoid-regulated kinase 1 (SGK1) is a member of the protein kinase A, G, and C (AGC) family (Firestone et al., 2003). SGK1 was first identified in rat mammary gland tumor cells in response to serum/glucocorticoid stimulation, and its expression has been detected in all tissues (Webster et al., 1993). Subsequently, several studies have identified that the expression and function of SGK1 are modulated by myriad factors, including hormones such as insulin, insulin-like growth factor 1, steroids, and cytokines such as interleukin-2 (IL-2) and transforming growth factor-β (TGF-β) (Webster et al., 1993; Perrotti et al., 2001; Amato et al., 2007; Boini et al., 2008; Lang and Voelkl, 2013).

Modulation of SGK1 requires both transcriptional and enzymatic regulation. As an initial step, extracellular stimuli bind to cognate receptors on the cell surface or in the cytosol. This process induces several transcription factors, such as glucocorticoid receptor, mineralocorticoid receptor (MR), and tumor suppressor p53, to migrate into the nucleus and they bind to specific sites within the promoter region of SGK1, thereby facilitating SGK1 transcription (Firestone et al., 2003). These extracellular stimuli also initiate the phosphoinositide-3 kinase (PI3K) pathway, which converts phosphatidylinositol 4,5-bisphosphate (PIP2) into phosphatidylinositol 3,4,5-triphosphate (PIP3) at the plasma membrane (Janku et al., 2018). PIP3 then recruits phosphoinositide-dependent protein kinase 1 (PDK1) and protein kinase B (AKT), subsequently activating mammalian target of rapamycin complex 2 (mTORC2) (He et al., 2021). Eventually, SGK1 is phosphorylated by PDK1 and mTORC2, activating the enzymatic function of SGK1 (Biondi et al., 2001).

As a serine/threonine kinase, SGK1 catalyzes the phosphorylation of numerous target proteins, including N-Myc downstream-regulated gene 1 (NDRG1), forkhead transcription factor 3a (FOXO3a), neuronal precursor cell expressed developmentally downregulated 4-2 (NEDD4-2), tuberous sclerosis complex 2 (TSC2), Unc-51 like autophagy activating kinase 1 (ULK1), and β-catenin (Cicenas et al., 2022). Given its wide range of targets, SGK1 has been shown to affect multiple biological events, such as ion channel activity, proliferation, survival, and apoptosis, which support the critical role of SGK1 in various diseases (Ghani, 2022). In the present review, we focus on providing current and in-depth evidence regarding the function of SGK1 in sodium transport, cancer progression, and immune modulation. Furthermore, we summarized the developmental status of SGK1 inhibitors, as well as their structures and potencies evaluated in pre-clinical experimental settings. Collectively, this review sheds light on the implications of SGK1 in biology and proposes SGK1 inhibitors as potential drugs for clinically relevant diseases.

## 2 SGK1 structure and functions

### 2.1 Structure of SGK1

**FIGURE 1:**
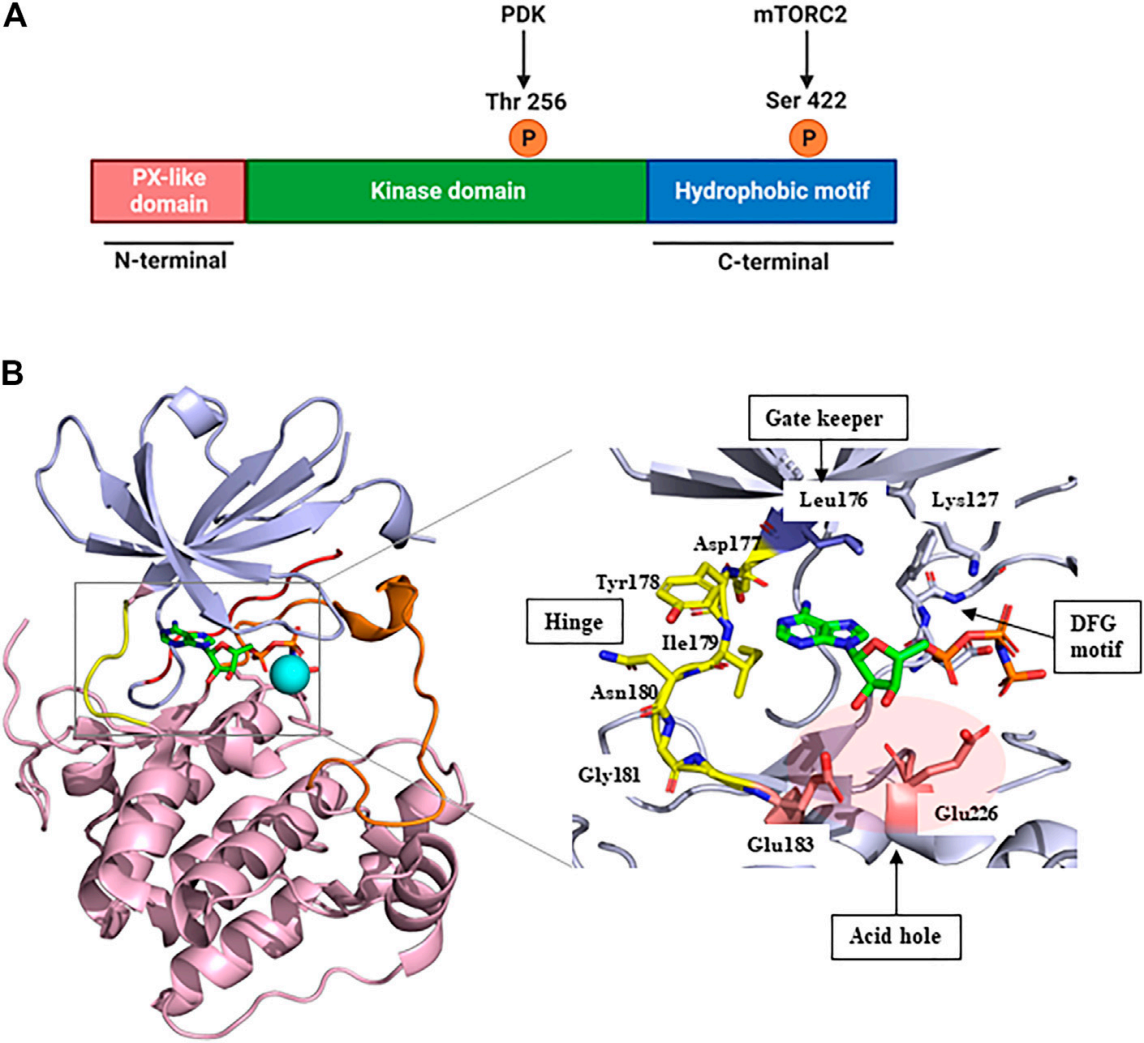
Structure of SGK1. **(A)** The protein domain structure of SGK1. SGK1 consists of an N-terminal PX-like domain, a kinase domain, and a C-terminal hydrophobic motif. The activity of SGK1 is governed by 2-step phosphorylation: 1) Extracellular stimuli induce mTORC2 activation, which phosphorylates Ser 422 at the hydrophobic motif. 2) PDK1 is recruited to the phospho-Ser, and transfers phosphate group to Thr 256 at the kinase domain. This figure was created using BioRender. **(B)** Three-dimensional (3D) structure of SGK1 kinase domain (left) and the details of ATP-binding site (right). mTORC2, mammalian target of rapamycin complex 2; PDK1, phosphatidylinositide-dependent protein kinase 1; SGK1, serum and glucocorticoid-regulated kinase 1.

SGK1, a member of the SGK family, comprises a kinase domain, a N-terminal phox homology domain, and a C-terminal hydrophobic motif (Figure 1A) (Basnet et al., 2018). To be comprehensively activated, SGK1 needs to undergo phosphorylation at specific sites of both the kinase domain and hydrophobic motif (Frodin et al., 2002). In response to growth factor stimulation, mTORC2 phosphorylates the hydrophobic motif of SGK1 at serine 422, inducing a conformational change to recruit PDK1 (Garcia-Martinez and Alessi, 2008). Subsequently, PDK1 phosphorylates the kinase domain of SGK1 at threonine 256 (Biondi et al., 2001; Lu et al., 2010). Accordingly, SGK1 is activated to modulate the functions of downstream genes through these phosphate transfer processes (Sang et al., 2020).

In 2007, Zhao et al. (2007) reported the crystal structure of an inactive SGK1 kinase domain complexed with the ATP analog ANP-PNP (Figure 1B). The kinase domain of SGK1 shares 45%–60% sequence homology with other serine/threonine protein kinases, such as cAMP-dependent protein kinase (PKA), the protein kinase C family, and AKT (Firestone et al., 2003). Overall, the SGK1 kinase domain structure has a typical protein kinase folding pattern consisting of β-strand structures at the *N*-terminus and α-helical structures at the *C*-terminus. The catalytic Lys127 and DFG motifs are conserved in the SGK1 kinase domain. In this co-crystal structure, the αC-helix domain was atypically unfolded, given that this crystal structure was an inactive conformation, resembling the conformation of inactive AKT (Huang et al., 2003) and catalytic Glu146 was lacking. The hinge moiety connecting the *N*- and *C*-termini comprises Asp-Tyr-Ile-Asn-Gly, and the gatekeeper residue is Leu176, a bulky amino acid. SGK1 also had an acid hole consisting of two glutamic acid residues (Glu183 and Glu226) at the lip of the ATP-binding site.

### 2.2 SGK1-mediated regulation of epithelial sodium channel

**FIGURE 2:**
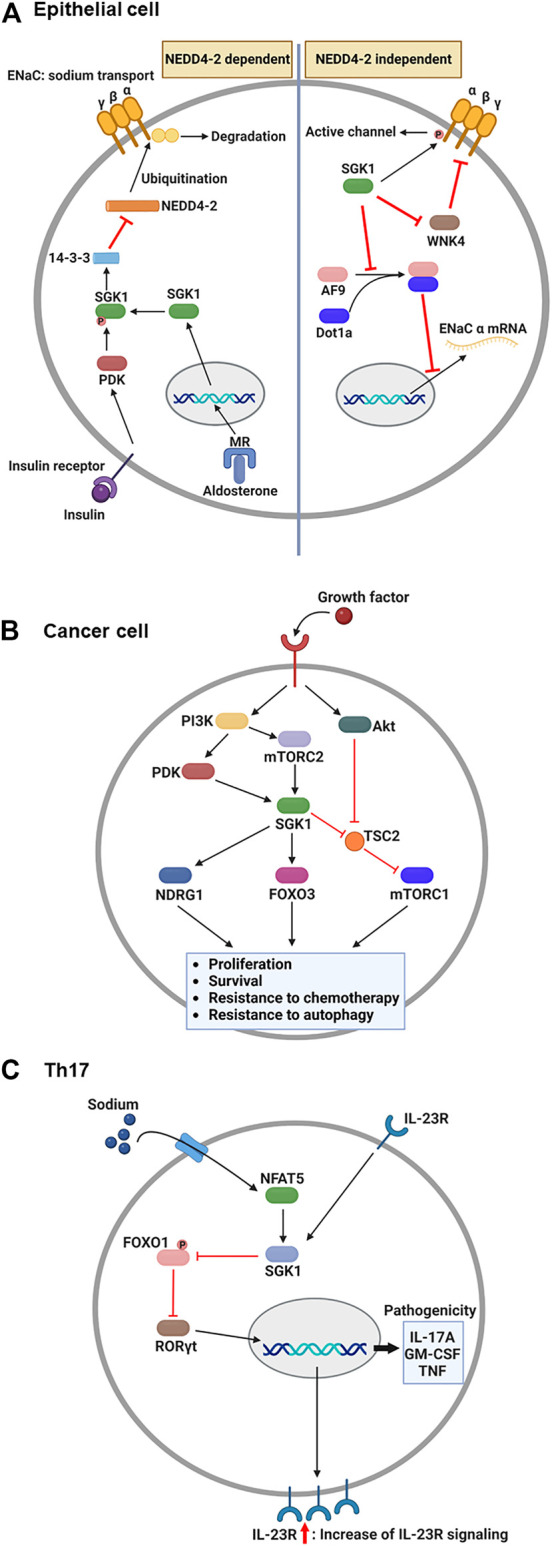
Overview: Role of SGK1 in biology. **(A)** Upon the aldosterone binding, cytosolic MR translocates into the nucleus and induces the transcription of SGK1 in renal epithelial cells. Concurrently, growth factors, such as insulin, activate PDK1, which then phosphorylates SGK1. Phosphorylated SGK1 inhibits NEDD4-2-mediated ubiquitination, which prevents the degradation of ENaC subunit. Additionally, SGK1 affects the function of ENaC in NEDD4-2 independent pathways. SGK1 directly phosphorylates ENaC *α* subunit to trigger channel activity. Moreover, SGK1 impedes a negative regulator of ENaC, WNK4, by which ENaC remains active for sodium reabsorption. Lastly, SGK1 promotes ENaC α transcription through restricting the formation of histone methyltransferase, AF9-Dot1a complex. **(B)** Upon growth factor-mediated stimulation, PI3K activates PDK1 and mTORC2, which in turn phosphorylates SGK1 in cancer cells. The enzymatic function of SGK1 turns on several target genes, such as NDRG1, FOXO3, and mTORC1, enhancing the cellular processes that facilitate cancer progression. **(C)** During Th17 differentiation, sodium exposure promotes NFAT5 and SGK1 activation. The enzymatic function of SGK1 hinders FOXO1 activity, which relieves RORγt from FOXO1-mediated inhibition. Subsequently, RORγt facilitates the expression of IL-23R, contributing to the enhancement of IL-23 signaling. As a positive feedback regulation, IL-23 promotes the SGK1-RORγt signaling pathway, resulting in the expression of pathogenic cytokines. ENaC, epithelial sodium channel; FOXO3, forkhead transcription factor 3; IL-23, interleukin-23; MR, mineralocorticoid receptor; mTORC1/2, mammalian target of rapamycin complex 1/2; NDRG1, N-Myc downstream-regulated gene 1; PDK1, phosphatidylinositide-dependent protein kinase 1; PI3K, phosphoinositide 3-kinase; SGK1, serum and glucocorticoid-regulated kinase 1. This figure was created using BioRender.

The epithelial sodium channel (ENaC) is necessary for sodium homeostasis in various epithelial tissues (Pearce, 2003). Hormone and non-hormone-dependent mechanisms regulate the activity of ENaC (Bhalla and Hallows, 2008). Upon hormonal regulation in aldosterone- and insulin-sufficient environment, NEDD4-2, an E3 ubiquitin ligase, plays a critical role in ENaC-mediated sodium reabsorption (Rotin and Staub, 2012) (Figure 2A). NEDD4-2 binds to the C-terminus of ENaC and ubiquitinates lysine residues on ENaC subunits (Flores et al., 2003), leading to a reduced density of ENaC on the cellular membrane owing to protein internalization and degradation (Butterworth, 2010). Thus, inhibition of NEDD4-2 function is an essential prerequisite for normal ENaC activity. Under stress conditions such as low blood volume/pressure and sodium-potassium imbalance, aldosterone is produced by the adrenal gland and binds to the MR in the cytosol of epithelial cells (Remuzzi et al., 2008). This nuclear hormone receptor then translocates to the nucleus to induce SGK1 transcription (Chen et al., 1999). The functional activation of SGK1 requires further phosphorylation. Upon insulin stimulation, PI3K stimulates PDK1, which phosphorylates SGK1 to become functional (Zhang et al., 2007). Then, active SGK1 phosphorylates NEDD4-2 to which 14-3-3 protein is recruited, preventing ENaC from NEDD4-2-mediated ubiquitination (Bhalla et al., 2005).

Besides, SGK1 can regulate the function of ENaC regardless of the presence of NEDD4-2 (Valinsky et al., 2018). Direct phosphorylation of ENaC is one of those NEDD4-2-independent channel modulation by SGK1 enzymatic activity. In *Xenopus laevis* oocyte system, SGK1-induced ENaC currents were impeded by C-terminus deletion of ENaC α subunit, but maintained in the truncation of either β or γ subunit (Diakov and Korbmacher, 2004). The channel activity was subsequently abrogated when the SGK1 consensus motif was mutated in ENaC α subunit, indicating that SGK1 directly modulates ENaC function. SGK1 also affects ENaC activity through controlling the function of another kinase, no lysine kinase 4 (WNK4). By targeting PY motif in the β and γ subunits of ENaC, WNK4 inhibited sodium reabsorption in normal physiology, but WNK4 mutation elevated sodium currents in *Xenopus laevis* oocytes and the distal colon of mouse (Ring et al., 2007a). A following study revealed that WNK4 has a conserved site for SGK1-mediated phosphorylation and WNK4 mutation that mimics SGK1 phosphorylation alleviates ENaC inhibition in *Xenopus* oocytes (Ring et al., 2007b). Furthermore, SGK1 transcriptionally regulates the expression of ENaC α subunit. Combining with disruptor of telomeric silencing alternative splice variant a (Dot1a), ALL1-fused gene from chromosome 9 (AF9) can function as a histone methyltransferase inhibiting mRNA expression of ENaC α subunit (Zhang et al., 2006). AF9 no longer bound to Dot1a when it became phosphorylated by SGK1, consequently relieving ENaC α from the repression of Dot1a-AF9 complex (Zhang et al., 2007).

Among ENaCs in multiple organs, a prominent role has been noted in epithelial cells in the distal tubules of the kidney. By reabsorbing sodium in the kidney, ENaC affects intra- and extracellular sodium levels, as well as fluid movement, consequently modulating fluid volume and blood pressure (Hanukoglu and Hanukoglu, 2016). Clinically, ENaC has been mutated to enhance sodium reabsorption, leading to hypertension due to fluid expansion in patients with Liddle syndrome (Enslow et al., 2019). Likewise, hyperaldosteronism mimics the continuous activation of ENaC and causes hypertension as an outcome of MR-driven SGK1 induction (Resch et al., 2010).

### 2.3 Tumor-promoting effect of SGK1

Over the last decade or more, the role of SGK1 in cancer has been demonstrated by several studies. Although few reports have noted SGK1 reduction and its correlation with poor prognosis in adrenocortical carcinoma (Ronchi et al., 2012) most studies have demonstrated that multiple types of tumors, such as colon, breast, and prostate cancer, exhibit higher levels of SGK1 expression than normal tissues (Sommer et al., 2013; Xiaobo et al., 2016; Liu et al., 2017; Lee et al., 2019). General alterations in the local/systemic environment, including nutrient availability, hormones, and growth factors, lead to SGK1 induction during tumor progression (Lang and Stournaras, 2013). As a family of serine/threonine kinases, SGK1 phosphorylates diverse signaling proteins other than NEDD4-2 and plays multifaceted roles in tumorigenesis, including survival, proliferation, apoptosis, and metastasis (Sang et al., 2020; Zhu et al., 2020).

Although the molecular mechanisms that drive tumorigenesis are still under investigation, the signaling pathways related to PI3K and mammalian target of rapamycin (mTOR) have been implicated as one of potential targets for antitumor therapy (Janku et al., 2018). AKT is considered the core component that mediates PI3K-induced signaling, consequently activating mTOR complex 1 (mTORC1) to induce protein synthesis required for tumor cell proliferation and survival (Bhutani et al., 2013). However, accumulated evidence indicates that SGK1 is an alternative downstream effector of the PI3K signaling pathway (Figure 2B). Another PI3K-stimulated mTOR subunit, mTORC2, phosphorylates the hydrophobic motif of SGK1 (Garcia-Martinez and Alessi, 2008), which induces a conformational change in SGK1. This change allows PDK1 to phosphorylate SGK1 for maximum activation (Kobayashi and Cohen, 1999; Talarico et al., 2016). SGK1 and AKT share several target genes to synergize the effect of PI3K signaling or compensate for the inhibition of either kinase (Di Cristofano, 2017). For example, Sommer et al. (2013) reported that resistance to AKT inhibitors was determined by elevated activation of SGK1 and NDRG1 in breast cancer cells and that AKT inhibitor-resistant cells ceased expanding when SGK1 was silenced *via* shRNA-induced knockdown. Furthermore, Castel et al. (2016) revealed that mTORC1 remained activated upon AKT suppression by a small-molecule inhibitor, BYL719, in breast cancer cells, owing to TSC2 phosphorylation by SGK1.

Furthermore, SGK1 modulates the proapoptotic function of FOXO3a. The functional regulatory sites of FOXO3a are phosphorylated by SGK1, which impedes transcriptional activity of FOXO3a *via* cytosolic localization. In fibroblasts, mutation of those phosphorylation sites inhibited BrdU incorporation, indicating unregulated FOXO3a activity enhanced cell cycle arrest. Additionally, mutant fibroblasts exhibited an increase of PARP cleavage that correlates with apoptosis (Brunet et al., 2001). These results suggest that SGK1 negatively regulates cell death through inhibiting FOXO3a activity. Anti-apoptotic function of SGK1 is also supported that FOXO3a is a critical regulator of apoptotic genes such as PUMA and BIM (You et al., 2006). FOXO3a also facilitates autophagy-mediated cell death under conditions exceeding the threshold for apoptosis (Chang and Zou, 2020). In prostate cancer cells, SGK1 enhanced FOXO3a accumulation in the cytosol, preventing the FOXO3a-induced transcription of autophagy-related genes (Liu et al., 2017). SGK1 knockdown resulted in FOXO3a dephosphorylation and restored LC3-mediated autophagy formation, subsequently inhibiting the growth of cancer cells (Liu et al., 2017).

Normal cells are prone to cell death in the conditions where stress signal is overly imposed on cells, but tumor cells are resistant to stress-induced cell death and even become progressive by stress signal (Eckerling et al., 2021). It has been elucidated that SGK1 has a stress-relieving effect that promotes the survival of tumor cells. In multiple myeloma cell line (MMCL), SGK1 overexpression reduced drug susceptibility to bortezomib that induces endoplasmic reticulum (ER) stress-driven cell death (Hoang et al., 2016). Following genomic analysis revealed that low SGK1 expression was correlated with favorable outcome in multiple myeloma patients treated with bortezomib, indicating that SGK1 promotes tumor growth *via* raising resistance to ER stress. Similarly, SGK1 showed a protective role in doxorubicin-induced cell death of cardiomyocytes (Wang and Han, 2022). Doxorubicin-treated cardiomyocytes displayed the elevated levels of GRP78, PERK, ATF4, and CHOP, which leads to ER stress-driven cell death. However, SGK1 overexpression attenuated ER stress and restored cell viability, supporting a significant role of SGK1 in modulating ER stress.

### 2.4 SGK1-mediated immune modulation

Although genetic factors are critical for immune modulation, it has been reported that external factors play indispensable roles in the elaborate regulation of the immune system (Brodin et al., 2015). Diet is an external factor that shapes the immunological condition, as diet-derived nutrients or metabolites bind to their cognate receptors in immune cells to initiate molecular programs for development, activation, and differentiation (Maslowski and Mackay, 2011; Kedia-Mehta and Finlay, 2019). A westernized diet can lead to an elevated incidence of multiple diseases, including cardiovascular diseases, diabetes, and autoimmune diseases (Ezzati and Riboli, 2013; Christ et al., 2019). Two key factors modulate the impact of westernized diets on disease occurrence: fat and salt (Statovci et al., 2017; Wilck et al., 2019). Herein, we review the mechanisms through which salt exacerbates autoimmune diseases *via* SGK1-induced signaling in CD4^+^ T cells. Additionally, we discuss the mechanisms by which SGK1 shapes the fate of macrophages regardless of salt.

Depending on cytokines present during T cell activation, antigen-stimulated CD4^+^ T cells differentiate into a specific subset to protect the host from pathogens (Jiang and Dong, 2013). However, under the combination of certain genetic and environmental factors, inflammatory CD4^+^ T cells are known to accumulate abnormally, expediting the progression of autoimmune diseases (O'Shea and Paul, 2010). One such inflammatory T cell subset is the interleukin (IL)-17-producing CD4^+^ T cells, the so-called Th17 (Yasuda et al., 2019). Regarding the association between salt and Th17, notable studies from two independent groups have identified that high salt directly stimulates the generation of autoimmune Th17 cells (Figure 2C). The first study by Kleinewietfeld et al. (2013) reported that increased salt levels enhanced Th17 differentiation from naïve CD4^+^ T cells. Microarray analysis revealed that salt-treated Th17 cells displayed pathogenic gene signatures, including abundant transcripts of IL-17A, IL-23R, and granulocyte-macrophage colony-stimulating factor (GM-CSF). Knockdown assays have shown that the salt-driven pathogenic program in Th17 cells is dependent on NFAT5 and SGK1. Additionally, Wu *et al.* analyzed genome-wide transcripts of *in vitro* polarized Th17 cells (Wu et al., 2013), identifying *Sgk1* as the most highly expressed gene. IL-23 receptor deficiency reduced the expression of SGK1 in Th17 cells, indicating that IL-23 signaling governs SGK1 induction in Th17 cells. The activated enzymatic function of SGK1 could inhibit FOXO1 activity. This step relieves RORγt from FOXO1-mediated inhibition, which upregulates IL-23R expression in Th17 cells to efficiently consume IL-23. Using an experimental autoimmune encephalomyelitis model, both studies demonstrated that high salt diet-fed mice exhibited more severe symptoms than normal diet-fed mice, accompanied by elevated Th17 accumulation in the central nervous system (Kleinewietfeld et al., 2013; Wu et al., 2013).

Recently, additional mechanisms through which SGK1 aggravates autoimmunity have been elucidated and are linked to regulatory T cells (Tregs). Tregs are a subset of CD4^+^ T cells with immunosuppressive functions that maintain immune homeostasis (Lucca and Dominguez-Villar, 2020). Foxp3 plays a critical role in Treg development (Sakaguchi et al., 2008). Wu et al. (2018) revealed that the elevated IL-23 signal by SGK1 could suppress Foxp3 expression, which preferentially converts CD4^+^ T cells into Th17 cells, but not Treg cells. Furthermore, Yang et al. (2020) reported that high salt levels induce Tregs to express SGK1, resulting in the generation of RORγt-expressing Tregs that exhibit an inflammatory phenotype (Ohnmacht et al., 2015).

In aspect of innate immunity, macrophages play an indispensable role in defending hosts from pathogens, but uncontrolled activation rather results in deleterious outcome. Macrophages produce inflammatory cytokines by recognizing pathogen-derived substances, such as lipopolysaccharide (LPS), peptidoglycan, and flagellin, of which process is tightly regulated by both intrinsic and extrinsic factors (Murray and Wynn, 2011). Notably, SGK1 has been reported to coordinate macrophage activation. Yang et al. (2012) demonstrated that SGK1 knock-out alleviated pathologic lesion in mouse model of angiotensin II (Ang II)-induced cardiac fibrosis, which was attributed to STAT3-dependent activation/infiltration of macrophage. In parallel, a recent study showed that inhibiting SGK1 with chemical inhibitor mitigated the severity of Ang II-induced cardiac fibrosis indicating the potential of SGK1-targeting strategy to cure inflammatory diseases. Moreover, a SGK1 inhibitor suppressed the levels of IL-1β in Ang II-treated mice and bone marrow-derived macrophages (BMDM), of which effect disappeared by NLRP3 siRNA transfection (Gan et al., 2018). This result suggests that SGK1 can exacerbate cardiac fibrosis through activating NLRP3 inflammasome.

In other experimental setting, several studies have demonstrated that SGK1 negatively modulates toll-like receptor (TLR)-mediated inflammation. Ren et al. examined the effect of SGK1 in both classically- and alternatively-activated macrophages by using pharmacological inhibitor and conditional knock-out mice (Ren et al., 2021). Restraining SGK1 augmented FOXO1-mediated production of inflammatory cytokine in classically-activated macrophages while reduced STAT3-mediated immunosuppression in alternatively-activated macrophages. Likewise, Han et al. (2022) demonstrated that SGK1 has an inhibitory effect in bacteria-induced inflammation. In *Porphyromonas gingivalis* (Pg)-stiumulated BMDM, SGK1 inhibition enhanced the production of inflammatory cytokines (TNF-α, IL-6, and IL-1β) while decreased the expression of immunosuppressive IL-10. Moreover, the authors showed that SGK1 limited TRAF2 phosphorylation by using LysM-Cre x *Sgk1* ^fl/fl^ mice, which in turn maintained TRAF3 activity that can restrict the function of NFκB. In this context, SGK1 inhibition also aggravated bone loss in Pg-infected mice, possibly suggesting that SGK1 is a negative regulator of inflammation in infection.

## 3 Targeting SGK1 using small-molecule inhibitors

### 3.1 SGK1 inhibitors related to sodium transport regulation

#### 3.1.1 EMD638683 (**1**)

In dietary fructose- or fat-induced hyperinsulinemic mice, SGK1 could improve renal tubular Na^+^ reabsorption and account for blood pressure elevation following a high-salt diet. Based on this observation, EMD638683 (**1**), an SGK1 inhibitor, was characterized as an antihypertensive agent by Ackermann et al. (2011). EMD638683 is an *N*′-homobenzoyl benzohydrazide analog which is an atypical scaffold in kinase inhibitors. Biochemically, EMD638683 selectively inhibited SGK1 over 69 kinases, including SGK2, SGK3, and AGC family members (Table 1).

**TABLE 1:** Small-molecule serum and glucocorticoid-regulated kinase 1 (SGK1) inhibitors.

Compound (original code)	Structure	SGK1 inhibition	Target disease tested	Biological effect	Ref
**1 (EMD638683)**	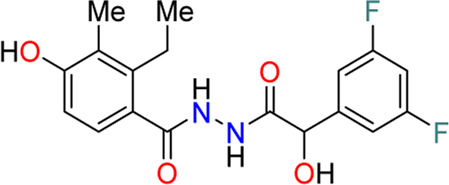	15% residual SGK1 activity at 1 µM	Hypertension/Inflammation	■ IC_50_ = 3.35 ± 0.32 µM (HeLa cells, p-NDRG1)	Sherk et al. (2008)
				■ Blood pressure = (HeLa cells, p-NDRG1)	Ackermann et al. (2011)
**2 (5377051)**	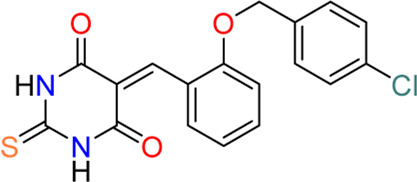	IC_50_ = 2.1 µM (FP assay)	Prostate cancer/Cardiac arrhythmia	■ Reduced p-NDRG1 (0.5–100 μM, LNCaP)	Bezzerides et al., 2017
				■ 80%–90% reduction in I_Na_ (10 μM, HEK293 with SGK1 S422D)	Liang et al. (2017)
**3 (Compound 1)**	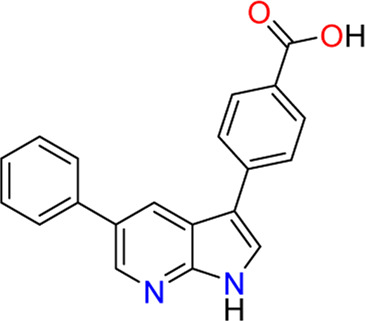	IC_50_ = 40 nM (FP assay)	N/A	■ IC_50_ = 1.3 µM (M-1 cells, SCC assay)	Hammond et al. (2009)
**4 (Compound 2)**	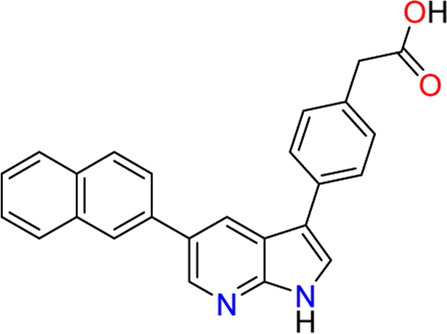	IC_50_ = 63 nM (FP assay)	N/A	■ IC_50_ = 0.88 µM (M-1 cells, SCC assay)	Hammond et al. (2009)
**5 (PO-322)**	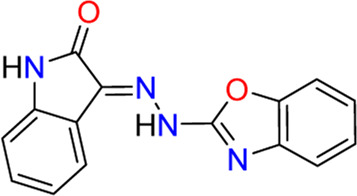	IC_50_ = 54 ± 6 nM (FP assay)	immunosuppressant	■ IC_50_ = 0.7 ± 0.2 μM (human T cell proliferation after anti-CD3 or anti-CD28 stimulation)	Ackermann et al. (2011)
				■ IC_50_ = 0.6 ± 0.3 μM (human T cell proliferation after alloantigen stimulation) Reduced p-NDRG1	Lai et al. (2020)
**6 (GSK650394)**	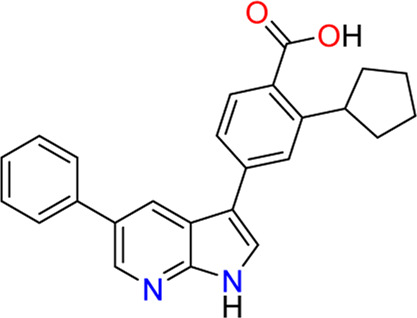	IC_50_ = 62 nM (*in vitro* activity-based scintillation proximity assay	Prostate Cancer	■ LC_50_ = 41 μM (M-1 cells, XTT assay)	Sherk et al. (2008)
				■ LC_50_ > 100 μM (M-1 cells, XTT assay)	
				■ IC_50_ = 0.6 μM (SCC assay)<	
				■ IC_50_ = ∼1 μM	
				■ (androgen-stimulated LNCaP cells)	
**7 (QGY-5-114-A)**	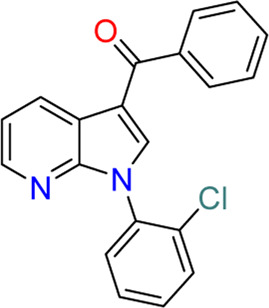	N/A	Colorectal Cancer	■ IC_50_ = 122.9 μM (HCT116 cells, CCK-8 assay)	Bezzerides et al. (2017)
				■ Impaired migration of HCT116 cells (transwell migration assay)	Liang et al. (2017)
**8 (SI113)**	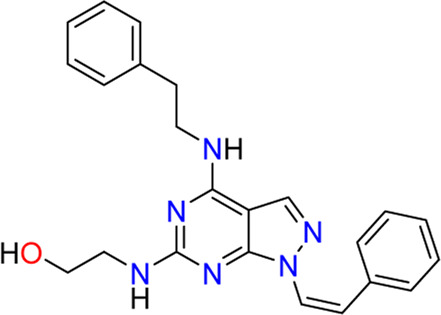	0% residual SGK1 activity at 12.5 µM	Cancers, including glioblastoma Multiforme	■ IC_50_ = 8 µM (RKO cells)	D'Antona et al. (2015); Lai et al. (2020)
				■ IC_50_ = 11.2 µM (LI cells)	
				■ IC_50_ = 10 µM (ADF cells)	
				■ IC_50_ = 9.1 µM (LI cells)	
				■ [All: cell viability assay]	
**9 (10) (Compound 14g)**	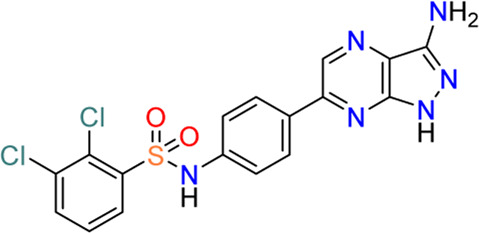	IC_50_ = 3 nM (10 μM ATP) IC_50_ = 442 nM (500 µM ATP)	Alpelisib-resistant breast cancer	■ EC_50_ = 1.4 μM (GSK3β phosphorylation, U2OS cells)	Halland et al. (2015); Castel et al. (2016)
				■ Reduced p-NDRG1 (HCC1954 and JIMT1 cells; combined with alpelisib)	
				■ Reduced cell viability and tumor burden (HCC1954 cells or xenograft mice; combined with alpelisib)	
**10 (Compound 7a)**	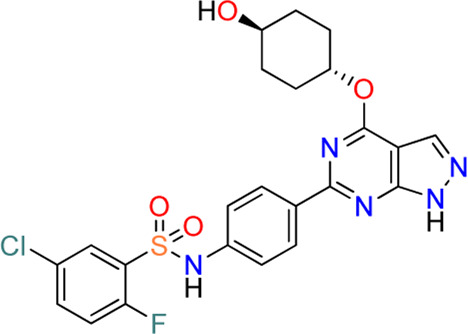	IC_50_ = <1 nM (10 µM ATP)IC_50_ = 3 nM (500 µM ATP)	osteoarthritis pathology	■ IC_50_ = 50 μM (ATDC5 cells; collagen type X expression)	Halland et al. (2022)
				■ Reduced collagen type X (2 μM, a mouse femoral head *ex vivo* model)	
**11 (Herbacetin)**	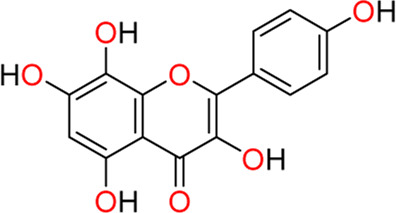	IC_50_ = 0.75 µM (FP assay) K_d_ = 0.84 µM (KINOMEscan)	Myocardial hypertrophy	■ Reduced p-FoxO1 and p-SGK (PE-treated cardiomyocyte)	Zhang et al. (2022)
				■ Cardioprotection	
				■ (mice treated with ISO, 10 mpk)	

FP, fluorescence polarization; ISO, isoproterenol; PE, phenylephrine; SSC, short circuit current.

EMD638683 inhibited the phosphorylation of NDRG1 in HeLa cells, presenting an IC_50_ value of 3.35 ± 0.32 µM. The *in vivo* efficacy of EMD638683 in SGK-dependent hypertension was further evaluated using a mouse model which was pretreated with fructose and saline to substantially increase blood pressure. A 4-day administration of EMD638683 could normalize the systolic blood pressure from 111 ± 4 to 87 ± 3 mmHg.

Infusing Ang II into mice induced renal/cardiac dysfunction with an elevated Th17/Treg ratio in relevant tissues when compared with that in control mice (Du et al., 2018). Moreover, a notable increase in the expression of SGK1 transcript was documented, along with the phosphorylation of SGK1 protein; treatment with the SGK1 inhibitor EMD638683 reversed Ang II-induced phenotypes to normal levels.

#### 3.1.2 5377051 (**2**)

Using *in silico* drug screening with an *in vitro* kinase assay, Bezzerides et al. (2017) discovered 5377051 (**2**), a thioxodihydropyrimidine-4,6-dione analog, as an SGK1 inhibitor. After constructing an SGK1 homology model based on structurally closed proteins and modification using the SGK1 crystal structure (PDB code: 2R5T), the authors screened approximately 500,000 small molecules to identify approximately 50 hits with eight major chemotypes, presenting IC_50_ values in the low micromolar to the sub-micromolar range. Finally, 5377051, with an IC_50_ value of 2.1 µM *in vitro* kinase assay, was selected as the lead compound. This compound (5377051) was validated in the LNCaP prostate cancer cell line. Reportedly, 5377051 reduced SGK1-mediated phosphorylation of NDRG1, a known SGK1 substrate, and inhibited the proliferation of LNCaP cells; the proliferation of these cells was induced by treatment with R1881, a synthetic androgen. In addition, 5377051 could inhibit the phosphorylation of another SGK1 substrate, GSK3β, expressed in neonatal rat ventricular myocytes.

SGK1-mediated regulation of the cardiac voltage-gated sodium channel (Na_V_ 1.5) was evaluated using HEK293 cells expressing Na_V_ 1.5, along with co-expression of a constitutively active SGK1 mutant (SGK1 S422D) or an inactive SGK1 mutant, SGK1 K127M. SGK1 S422D-expressing HEK293 cells demonstrated a significant increase in sodium flux, whereas a significant decrease in sodium flux was detected in SGK1 K127M-expressed HEK293 cells (Bezzerides et al., 2017). Based on this observation, the effect of 5377051 on the modulation of sodium currents in HEK293 cells expressing SGK1 S422D was investigated. Treatment with 10 µM 5377051 markedly reduced sodium currents to levels observed in HEK293 cells expressing SGK1 K127M.

#### 3.1.3 Compounds 1 (**3**) and 2 (**4**)

**FIGURE 3:**
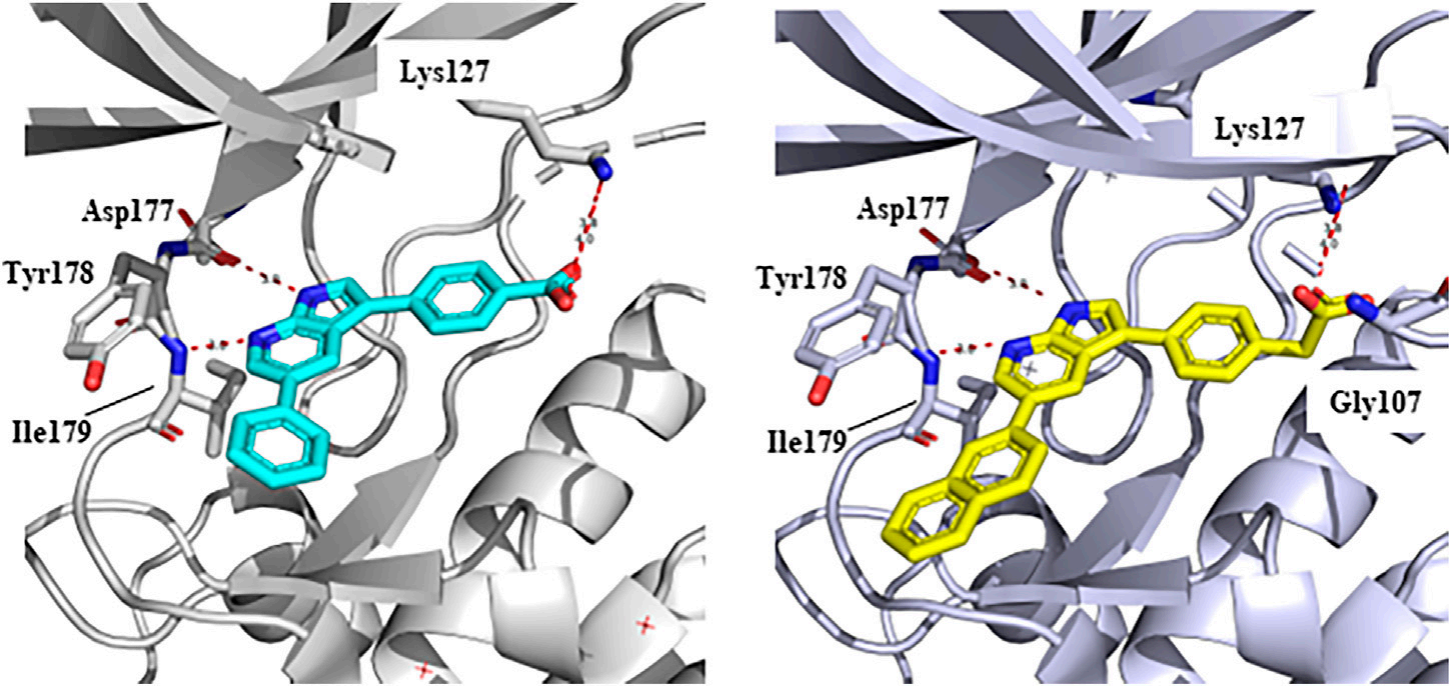
Co-crystal structures of SGK1 complexed with compound 1 (**3**) (left; PDB code: 3hdm) and 2 (**4**) (right; PDB code: 3hdn). Red dotted lines indicate hydrogen bonds between inhibitors and SGK1. SGK1, serum and glucocorticoid-regulated kinase 1.

In 2009, a research team at GSK pharmaceuticals led by M. Hammond identified azaindole-based SGK1 inhibitors as part of a program to study the role of SGK1 and reported their structure-activity and structure-property relationships (Hammond et al., 2009). Two hit compounds 1 (**3**) and 2 (**4**) displayed robust SGK1 inhibitory activity with two-digit nanomolar IC_50_ values (40 nM for compound 1 and 63 nM for compound 2) and approximately 1 µM IC_50_ values in a whole-cell M-1 SCC assay (1.3 µM for compound 1 and 0.88 µM for compound 2). Co-crystal structures of SGK bound to each hit compound showed that two nitrogen atoms of the azaindole core formed donor-acceptor hinge contacts at the ATP-binding site with a carbonyl of Asp177 and an NH of Ile179 (Figure 3). The carboxylates, connecting the para-position of the 3-phenyl substituent without a spacer in compound 1 or with a methylene spacer in compound 2, interact with catalytic lysine (Lys127) and/or the backbone nitrogen of Gly107. The authors proposed that these carboxylates might be responsible for the poor oral pharmacokinetic (PK) properties by either limiting absorption owing to their polar and hydrogen-bonding characteristics or favoring elimination owing to mechanisms such as glucuronic acid conjugation. Given that the electrostatic interactions of carboxylates with Lys127 are critical for SGK1 inhibition in this series, Hammond et al. (2009) modified the structures of two hits by incorporating a bulky substituent while maintaining the carboxylate group to improve rat PK properties (Table 2). Accordingly, compound 16 (**12**), an analog of compound 1 possessing an *ortho*-ethyl substituent, showed improved potency (SGK1 IC_50_ = 20 nM) and rat PK properties, in part, with lower plasma clearance (CLp = 9.4 ml/min/kg) and 4-fold improved dose normalized AUC_0-t_ (area under the concentration-time curve from 0 to time t; 600 ng h/mL/mg/kg) following oral administration, compared with compound 1 (CLp = 64.4 ml/min/kg; DNAUC_0-t_ = 154 ng h/mL/mg/kg). Compounds 20 (**13**) and 22 (**14**), analogs of compound 2, also showed improved PK properties, along with similar potencies to compound 2.

**TABLE 2:** *In vitro* SGK1 inhibition and rat PK properties of compounds 1, 2, and their analogs (Hammond et al., 2009).

Compound (original code)	Structure	Rat PK properties						
		SGK1 IC_50_ (nM)	M-1 SCC IC_50_ (µM)	CL_p_ (ml/min/kg)	Vd_ss_ (L/kg)	IV t_½_ (min)	Oral AUC (ng h/ml)	OralDNAUC (ng h/ml/mg/kg)
**3 (Compound 1)**	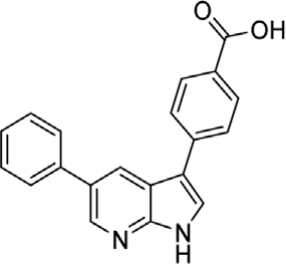	40	1.30	64.4	8.8	564	392	154
**4 (Compound 2)**	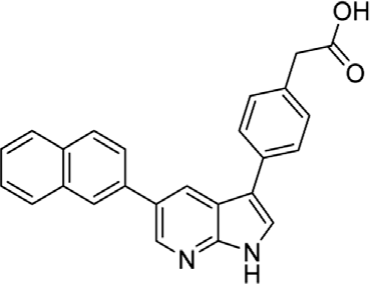	20	0.71	9.4	0.43	97	1,499	600
**12 (Compound 16)**	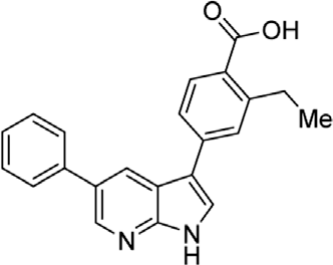	63	0.88	21.3	1.3	253	467	225
**13 (Compound 20)**	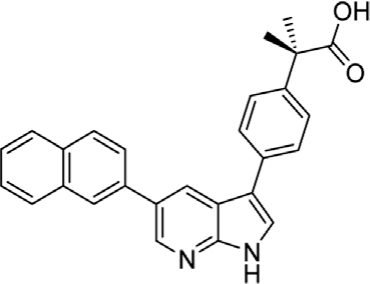	63	0.87	4.32	0.67	264	4,173	2,100
**14 (Compound 22)**	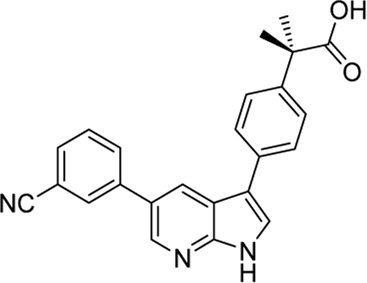	40	2.0	6.56	2.5	488	1913	905

AUC, area under the concentration-time curve; CL_p_, plasma clearance; DNAUC, dose normalized AUC; IV, intravenous; PK, pharmacokinetics; SGK1, serum and glucocorticoid-regulated kinase 1; SSC, short circuit current; Vd_ss_, volume of distribution at steady state.

### 3.2 SGK1 inhibitors as an immune modulator

#### 3.2.1 PO-322 (**5**)

Lai et al. (2020) identified PO-322 (**5**) as a selective SGK1 inhibitor that acts as an immunosuppressant. Initially, the authors examined the effects of in-house oxazole derivatives on T cell proliferation following anti-CD3 or anti-CD28 stimulation. The most potent inhibitor, PO-322, could inhibit human T cell proliferation after anti-CD3 or anti-CD28 stimulation, presenting IC_50_ values of 0.7 ± 0.2 µM and alloantigen stimulation with an IC_50_ value of 0.6 ± 0.3 µM. Moreover, PO-322 displayed no significant cytotoxicity against resting T cells, activated T cells, or peripheral blood mononuclear cells. Target kinases of PO-322 were identified using a kinase profiling assay with 100 kinases. PO-322 is a highly selective SGK1 inhibitor, affording 98% inhibition, and no other kinases showed >50% inhibition when treated with 1 µM concentration. The IC_50_ value of PO-322 against SGK1 cells was 54 ± 6 nM. PO-322 treatment reduced NDRG1 phosphorylation while increasing 70S6K and STAT5 phosphorylation in T cells, indicating that the effect of PO-322 on T cell proliferation is mediated *via* the SGK1/NDRG1 signaling pathway. PO-322 exhibited anti-inflammatory effects by inhibiting IL-17, interferon (IFN)-γ, and IL-6 expression. Furthermore, PO-322 effectively reduced the DTH reaction induced by DNFB and improved imiquimod-induced dermatitis in mice.

### 3.3 SGK1 inhibitors in cancers

#### 3.3.1 GSK650394 (**6**)

In 2008, Sherk et al. (2008) reported the first SGK1 inhibitor, GSK650394 (**6**). GSK650394 is a 7-azaindole derivative substituted with two aryl groups at C3 and C5 positions, and the 3-phenyl group has a carboxylic acid and cyclopentyl group at para- and meta-positions, respectively. The binding affinity of GSK650394 for activated SGK1, determined using a fluorescence polarization assay with rhodamine green-labeled ATP mimetics, presented an IC_50_ value of 13 nM. GSK650394 potently inhibited the enzymatic activity of SGK1 and SGK2, as measured by an *in vitro* activity-based scintillation proximity assay, with IC_50_ values of 62 and 103 nM, respectively. GSK650394 showed marginal cytotoxicity against M-1 cells (LC_50_ value = 41 µM) and HeLa cells (LC_50_ value > 100 µM) in XTT assays assessing mitochondrial enzymatic activity. Furthermore, the effects of GSK650394 on the SGK1-mediated regulation of epithelial sodium ion transport in response to aldosterone stimulation were evaluated using an aldosterone-stimulated short circuit current (SCC) cellular assay. GSK650394 displayed an IC_50_ value of 0.6 µM in this SCC assay. The selectivity of GSK650394 for SGK1, compared with the closely related AGC kinase family members, AKT, and other related kinases, was examined using *in vitro* kinase assays. GSK650394 displayed high selectivity for SGK1 when compared with these kinases, with more than 30-fold selectivity. However, a number of off-targets with potencies comparable to SGK1 were identified among 85 kinases examined by extensive selectivity profiling of GSK650394, provided by the MRC Protein Phosphorylation Unit, including AMPK, CAMKKβ, CDK2, GCK, MNK1, and PHK (Maestro et al., 2020). GSK650394 repressed androgen-mediated phosphorylation of NEDD4-2, a well-known SGK1 substrate, in LNCaP prostate cancer cells. In a 10-day cell growth assay, GSK650394 showed no effect on LNCaP cell growth in the absence of androgens but exhibited strong inhibition of the androgen-stimulated growth of LNCaP cells with an IC_50_ value of approximately 1 μM, indicating that GSK650394 selectively inhibits androgen receptor (AR)-driven prostate cancer cells, but not AR-liberated prostate cancer cells.

#### 3.3.2 GY-5-114-A (**7**)

Among 39 analogs of GSK650394, which were designed and synthesized by Liang et al. (2017) 7 compounds could inhibit the cell viability of colonic tumor cell line HCT116, as determined by CCK-8 assay; however, only one analog, QGY-5-114-A (**7**), showed a significantly lower IC_50_ value (122.9 µM) than that of GSK650394 (135.5 µM). Furthermore, QGY-5-114-A impaired the migration of HCT116 cells, as determined by the transwell migration assay, and reduced tumor growth *in vivo*, evaluated using nude mice subcutaneously inoculated with HCT116 cells. Although QGY-5-114-A and GSK650394 both contain azaindole cores, QGY-5-114-A has a 2-chlorophenyl group that masks the free NH of 7-azaindole, which forms a crucial hydrogen bond with the hinge backbone of SGK1, disclosed by the co-crystal structure of SGK1 complexed with other 7-azaindole SGK1 inhibitors (Hammond et al., 2009); this suggested that QGY-5-114-A and GSK650394 potentially exhibit distinct binding modes. Moreover, the entire biological assessment of QGY-5-114-A was conducted using cell-based assays rather than biochemical tests, hindering the direct comparison of QGY-5-114-A to GSK650394 for SGK1 inhibition.

#### 3.3.3 SI113 (**8**)

Screening of substituted pyrazolo [3,4-d]pyrimidine analogs, previously characterized as dual Src/Abl inhibitors against both SGK1 and AKT, resulted in the discovery of SI113 (**8**) as a selective SGK1 inhibitor when compared with Abl, Src, and AKT, which share structural and functional similarities with SGK1 (D'Antona et al., 2015). In cell-based assays, SI113 was determined as a potential antitumor agent. Treatment with 12.5 µM of SI113 was shown to reduce the *in vitro* cell growth of three different cancer cell lines (MCF-7, A-172, and RKO), and the dose-escalation assay against RKO exhibited an IC_50_ value of 8 µM. Furthermore, SI113 inhibited the phosphorylation of the SGK1 substrate MDM2 in the presence or absence of the SGK1 activator insulin.

In a follow-up study, the effect of SI113 was also evaluated in glioblastoma multiforme (GBM), one of the most aggressive brain tumors, given that SGK1 overexpression has been documented in GBM (Talarico et al., 2016). Treatment of three GBM cell lines (LI, ADF, and A172) with SI113 resulted in a significant reduction in cell viability with IC_50_ values of 11.2, 10, and 9.1 μM, respectively, with modest effects on normal fibroblasts. Although SI113 has shown good anticancer activity, there is potential for further improvement, given that the structure-activity relationship (SAR) has been poorly examined.

#### 3.3.4 *N*-(4-(1*H*-pyrazolo [3,4-*b*]pyrazin-6-yl)phenyl)benzenesulfonamides (**9**)

**FIGURE 4:**
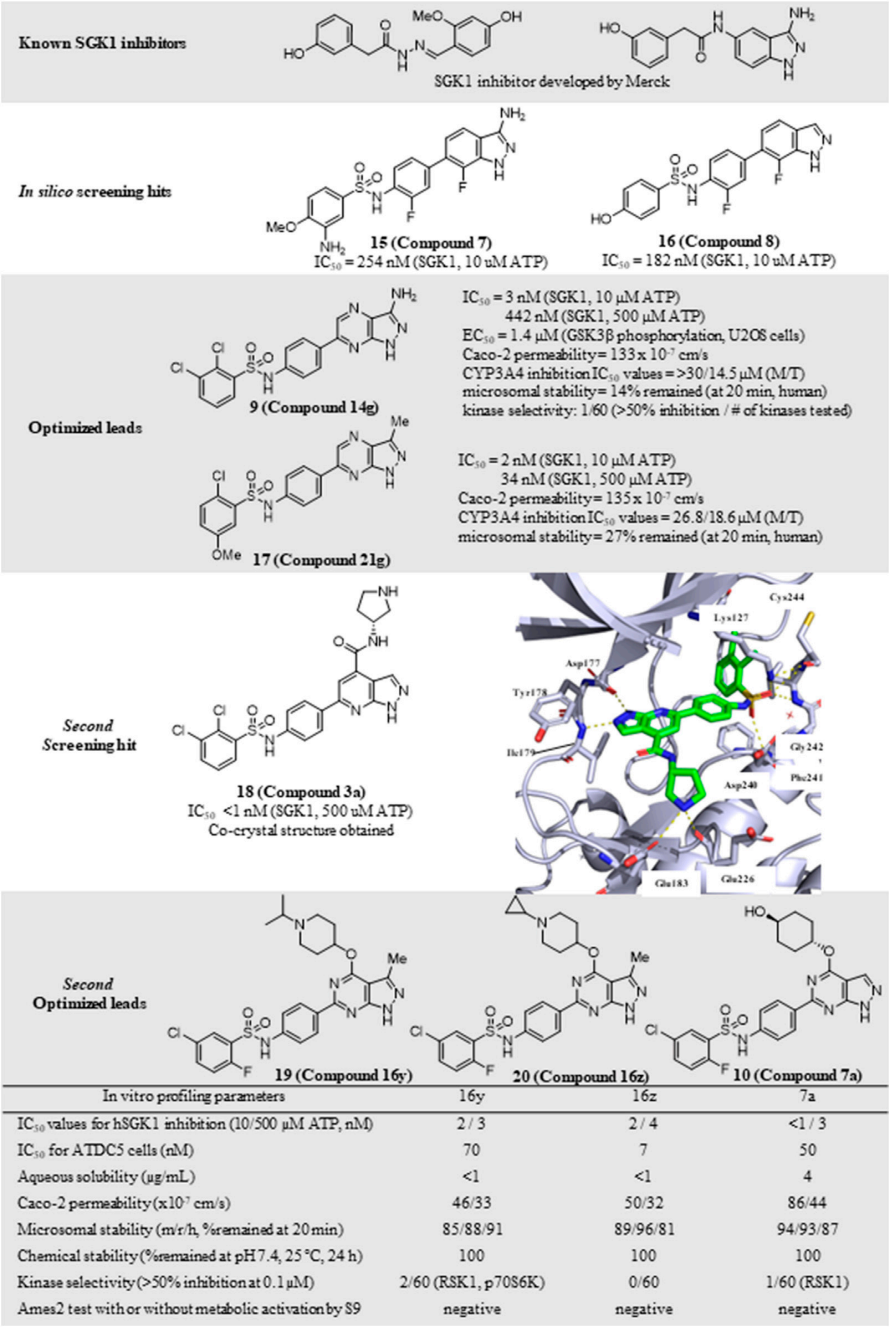
Discovery of *N*-(4-(1*H*-pyrazolo[3,4-*d*]pyrimidin-6-yl)phenyl)-5-chloro-2-fluorobenzenesulfonamides as an SGK1 inhibitor. SGK1, serum and glucocorticoid-regulated kinase 1.

Despite the well-established biological functions of SGK1 in relation to diverse diseases, the discovery of small-molecule SGK1 inhibitors with adequate selectivity and efficacy against SGK1 remains challenging. Recently, Halland et al. (2015) reported novel SGK1 inhibitors with high kinome and off-target selectivity (Figure 4). Three-dimensional (3D) ligand-based virtual screening was performed using approximately two million chemical libraries, and similarities in molecular shape and electrostatics were determined using known SGK1 inhibitors. In total, 78 hit compounds were evaluated for potential SGK1 inhibitory activity using a substrate phosphorylation lab-chip caliper assay. Finally, 6-sulfamidophenyl-3-aminoindazole analogs (**15**, **16**) showed good SGK1 inhibitory activities, affording IC_50_ values of 254 and 182 nM at 10 µM ATP, respectively, which were selected for further investigation. Iterative chemical library syntheses were performed to investigate the SAR. The SGK1 inhibitory activities of synthesized analogs were evaluated at ATP concentrations of 10 and 500 µM to determine the potency of these analogs in the presence of almost physiological levels of endogenous ATP. Aryl and heteroarylsulfonamide analogs displayed strong SGK1 inhibition, presenting nanomolar range IC_50_ values at an ATP concentration of 10 µM. Small substituents on the benzenesulfonamide moiety, such as halide, methyl, cyano, or methoxy groups, afforded potent SGK1 inhibition, and the 2,5-substitution pattern was typically preferred when compared with the corresponding 2,3-substitution. Although analogs containing the 3-aminoindazole hinge-contact motif generally showed strong SGK1 inhibitory activities, their aqueous solubility was low at <0.001 mg/ml (pH = 7.4, 25°C). To improve physicochemical properties, a 1*H*-pyrazolo [3,4-*b*]pyrazin-3-amine moiety was evaluated as a hinge-contact motif. These analogs exhibited SGK1 inhibitory activities that were comparable with those of the corresponding 3-aminoindazole analogs. 1*H*-pyrazolo [3,4-*b*]pyrazine analogs with methyl substituent at the 3-position, instead of amine functionality, were markedly potent SGK1 inhibitors with IC_50_ values of <15 nM at 10 µM ATP, whereas the corresponding unsubstituted analogs at the 3-position showed greatly reduced SGK1 inhibition (IC_50_ values >400 nM at 10 µM ATP). Replacement of the sulfonamide moiety with a secondary amide to reduce lipophilicity resulted in a dramatic loss of activity. Three 1*H*-pyrazolo [3,4-b]pyrazine analogs, including two 3-amino-substituted analogs, 14 g (**9**) and 14n, and one 3-methyl-substituted analog, 21 g (**17**), were selected for further *in vitro* profiling. All three analogs exhibited good *in vitro* Absorption, Distribution, Metabolism, and Excretion (ADME) profiles, including calculated parameters related to Lipinski’s rule of five, aqueous solubility at pH 5.0, metabolic stability assessed by microsomal stability, Caco-2 permeability, and CYP3A4 inhibition. Moreover, 14g and 14n showed excellent kinome selectivity against 60 kinases. Only the AMP-related kinase was inhibited by ˃ 50% at a 1 μM concentration. Compounds 14n and 21g were the most potent SGK1 inhibitors in this chemical series; however, 14g was selected for pharmacokinetic profiling in rats owing to the favorable *in vitro* profile. The maximal plasma concentration (C_max_) was 3.88 μg/ml observed 2 h after oral administration of 3 mg/kg, and the half-life was 3 h, indicating that 14g was highly stable in plasma and had a moderate clearance rate. The highest distribution of 14g were observed in the liver, followed by the kidney and brain, but the overall tissue distribution was low.

The PDK1-SGK1 axis has been characterized as a drug-resistant mechanism of the PI3Kα inhibitor alpelisib in breast cancer, in which mTORC1 is activated *via* direct phosphorylation and inhibition of TSC2, a negative mTORC1 regulator, by SGK1 (Castel et al., 2016). In HCC1954 and JIMT1 cells, both alpelisib-resistant cell lines, combination treatment with 14g and alpelisib could reduce NDRG1 phosphorylation and mTORC1 signaling, resulting in more effective suppression of cell viability in HCC1954 cells.

### 3.4 SGK1 inhibitors in other diseases

#### 3.4.1 *N*-(4-(1*H*-pyrazolo [3,4-*d*]pyrimidin-6-yl)phenyl)-5-chloro-2-fluorobenzenesulfonamides (**10**)

Additional chemical screening campaigns and structural biology efforts have led to the identification of a new benzenesulfonamide analog 3a (**18**), binding the co-crystal structure of SGK1 (Halland et al., 2022) (Figure 4). The unique sulfonamide group directs the DFG loop, which induces a twisted conformation in the backbone. This interaction leads to the placement of the dichlorobenzene group in a deep hydrophobic pocket generated by several lipophilic residues. Reportedly, 3a has a distinct structural features from those of 14g, 14n, and 21g, such as 1*H*-pyrazolo [3,4-*b*]pyridine as a hinge-contact moiety and the basic pyrrolidine group, forming a salt bridge to the side chain of Glu183 in the ribose pocket, which is connected to the 4-position of 1*H*-pyrazolo [3,4-*b*]pyridine *via* an arylamide functionality. Although 3a displayed strong SGK1 inhibition with an IC_50_ value of less than 1 nM at 500 µM ATP concentration, an additional optimization campaign was performed, given the poor kinome selectivity of 3a owing to the basic pyrrolidine interacting with acidic residues from the kinase ribose pocket. Moreover, arylamide derivatives typically exhibit poor permeability, and *ortho*-chloroarylsulfonamide analogs are promising phototoxophores. Finally, three optimized analogs, 16y (**19**), 16z (**20**), and 17a (**10**), were selected as lead compounds, which exhibited superior potency and good drug-like properties. To avoid genotoxicity caused by the 3-amino substituent at the bicycle cores (Chen et al., 2006), 16y and 16z had a 3-methyl-1*H*-pyrazolo [3,4-*d*]-pyrimidine, and 17a had a non-substituted 1*H*-pyrazolo [3,4-*d*]-pyrimidine as a hinge-contact moiety. To increase the membrane permeability, the arylamide functionality at 4-position was replaced with an ether linkage, connecting to a substituted piperidine for 16y and 16z or a 4-hydroxycyclochexyl group for 17a, which interact with Glu183 and Glu226. Moreover, 2-fluoro-5-chlorobenzene was selected as a deep hydrophobic pocket binder to eliminate phototoxicity.

These three leads exhibited strong SGK1 inhibition with IC_50_ values of ≤4 nM at 500 µM ATP concentration, accompanied by good Caco-2 permeability, chemical stability at pH 7.4, and microsomal stability. Furthermore, the off-target kinase selectivity of the three leads was assessed against 60 kinases. Reportedly 16y could inhibit RSK1 and p70S6K by ˃ 50% at 0.1 μM, and 17a inhibited RSK1; however, none of the 60 kinases tested was inhibited by 16z. All leads were negative in the Ames2 mutagenicity test, with or without metabolic activation, using S9. Drawbacks of these leads included low aqueous solubility and weak human ether-à-go-go-related gene (hERG) inhibition, as determined in the *in vitro* ADMET tests.

The potential applicability of lead compounds in osteoarthritis pathology was examined by undertaking a comparative analysis of RNA expression. Expression levels of SGK1 were increased in diseased osteoarthritis cartilage, during which articular chondrocytes attain an aberrant hypertrophy-like state and lose phenotypic stability. To evaluate the effects of these lead compounds on chondrocyte hypertrophic differentiation, Halland et al. used a disease-specific assay to measure the expression levels of collagen type X, a specific marker, in ATDC5 cells. Compounds 16y, 16z, and 17a strongly inhibited collagen type X expression, with IC_50_ values of 70, 7, and 50 nM, respectively. This effect was further examined in an *ex vivo* mouse femoral head cartilage explant model. SGK1 inhibitors were used to treat femoral heads collected from 6-week-old C57BI6 mice and cultured for 2 weeks with anabolic stimuli. Collagen type X was detected using immunohistochemical staining. Treatment with each lead compound at a concentration of 2 μM reduced the staining intensity of collagen type X. Collectively, 16y, 16z, and 17a controlled osteoarthritis pathology, which was well-correlated with the SGK1 inhibitory activities of the lead compounds.

#### 3.4.2 Herbacetin (**11**)

In 2022, Zhang et al. (2022) discovered herbacetin (**11**), a potential SGK1 inhibitor derived from the extracts of *Rhodiola* species, which has a long history as an herbal medicine in Europe and East Asia. Extracts of *Rhodiola* species have been employed to improve immunity, fight fatigue syndrome or aging, reduce altitude sickness or hypoxia-induced oxidative stress, and relieve symptoms of ischemic heart disease. The active ingredients of Rhodiola extracts include phenyl ethanoids, phenyl propenoids, monoterpene glycosides, and flavonoids. Zhang et al. used a mass spectrometry-based SGK1 kinase assay to detect and quantify phosphorylated substrate peptides from a known SGK1 substrate, glycogen synthase kinase-3 (GSK-3). In this assay, herbacetin, rhodiosin, and *p*-coumaric acid were identified as SGK1 inhibitors from *Rhodiola* extracts, exhibiting IC_50_ values of 0.75, 6.62, and 8.04 µM, respectively. The most potent ingredient, herbacetin (K_d_ = 0.84 µM), showed a 14-fold higher binding affinity to SGK1 than a known SGK1 inhibitor, EMD638683 (K_d_ = 12 µM), as determined using the KINOMEscan assay (Eurofins Discovery). As herbacetin is a flavonoid, the SGK1 inhibitory activities of 20 flavonoids structurally related to herbacetin were evaluated. One analog lacking a 5-hydroxyl group exerted the highest SGK1 inhibition (IC_50_ = 0.31 µM), but the absence of a hydroxyl group at 4`-, 8-, or 3-positions reduced SGK1 inhibition. Among the 28 human AGC kinases, herbacetin selectively inhibited SGK1 and SGK2 when compared with SGK3, PRKG2, GRK3, ROCK-1, PKAc*β*, and other AGC kinases. Activation of SGK1 has been associated with cardiac hypertrophy, in which increased phosphorylated SGK1 and phosphorylated FoxO1, a downstream target of SGK1, were observed following cardiomyocyte treatment with phenylephrine (PE), a cardiac hypertrophic stimulant. Treatment with herbacetin could reduce PE-induced hypertrophy, as assessed by direct measurement of the cell area after Alexa Fluor 488 phalloidin staining. The *in vivo* cardioprotective effect of herbacetin was examined in mice treated with isoproterenol (ISO), a hypertrophic agonist. After treatment with ISO for 21 days *via* subcutaneous injection, 10 mg/kg herbacetin was administered once daily. Cardiac performance in ISO-treated mice was effectively improved following herbacetin administration, as determined by evaluating the increased left ventricular ejection fraction, left ventricular fraction shortening, decreased left ventricular volume, and reduced heart weight and tibial length, which significantly increased heart hypertrophy. Collectively, herbacetin effectively attenuated the progress of cardiac hypertrophy both *in vitro* and *in vivo*.

## 4 Conclusion

As a serine/threonine kinase, SGK1 catalyzes the phosphorylation of numerous target proteins, including NDRG1, FOXO3a, NEDD4-2, TSC2, ULK1, and β-catenin (Cicenas et al., 2022). Given its wide range of targets, SGK1 has gained momentum in the field of drug discovery, as it is capable of modulating essential cellular processes, including ion channel activity, hormone release, T cell activation, and fundamental features of cells such as proliferation, survival, and apoptosis. Although ubiquitously expressed, transcription of SGK1 is highly regulated by various cellular events, such as hyperglycemia, ischemia, and cancer, and stimulated by glucocorticoids, mineralocorticoids, insulin, and TGFβ. Hence, dysregulated SGK1 has been associated with multiple diseases, such as hypertension, cancer, autoimmunity, and neurodegenerative disorders. In the present review, we summarized the biological functions of SGK1, focusing on three major roles, i.e., in ion channels, tumor promotion, and immune modulation.

The development of a first-in-class small-molecule kinase inhibitor remains challenging. One of the major hurdles is overall kinome selectivity, given that most kinases share similar ATP-binding pockets. Despite the validated biological functions of SGK1, which are highly associated with various diseases, only a few SGK1 small-molecule inhibitors have been discovered, and none have progressed to the clinical stage. Most SGK1 inhibitors have insufficient potency and whole-kinome selectivity. Considering azaindole analogs 1, 3, and 4, SGK1 inhibition was strong with two-digit nanomolar IC_50_ values. However, their whole-kinome selectivity may need to be improved, given that the azaindole hinge-contact moiety and carboxylic acid functionality, interacting with a catalytic lysine, have been widely employed in other kinase inhibitors. Structurally distinct SGK1 inhibitors, such as 5, 6, and 7, may afford good kinome selectivity, but these inhibitors exhibited weak SGK1 inhibitory activities with micromolar IC_50_ values. Pyrazolopyrazine- or pyrazolopyrimidine-based SGK1 inhibitors, recently reported by Halland *et al.*, have been thoroughly optimized, resulting in the generation of highly potent and relatively selective SGK1 inhibitors 20-22 (16y, 16z, and 17a). Co-crystal structures of the SGK1 kinase domain complexed with this series revealed a distinct deep hydrophobic pocket generated by a twisted conformation of the DFG motif and backbone. Information regarding this new hydrophobic pocket will help generate innovative design strategies for developing new SGK1 inhibitors. Additionally, targeted protein degradation strategies have emerged, including proteolysis targeting chimeras (PROTACs), molecular glue, and lysosome-based degradation techniques. The application of this new technology in SGK1 inhibition may provide novel concepts for developing highly selective and potent SGK1 inhibitors. Taken together with the validated biological functions of SGK1, a new generation of more selective and potent SGK1 inhibitors is needed to develop a first-in-human SGK1 inhibitor and validate the therapeutic potential of SGK1 targeting.
